# The *Anopheles gambiae* Odorant Binding Protein 1 (AgamOBP1) Mediates Indole Recognition in the Antennae of Female Mosquitoes

**DOI:** 10.1371/journal.pone.0009471

**Published:** 2010-03-01

**Authors:** Harald Biessmann, Evi Andronopoulou, Max R. Biessmann, Vassilis Douris, Spiros D. Dimitratos, Elias Eliopoulos, Patrick M. Guerin, Kostas Iatrou, Robin W. Justice, Thomas Kröber, Osvaldo Marinotti, Panagiota Tsitoura, Daniel F. Woods, Marika F. Walter

**Affiliations:** 1 Developmental Biology Center, Department of Molecular Biology and Biochemistry, University of California Irvine, Irvine, California, United States of America; 2 Insect Molecular Genetics and Biotechnology Group, Institute of Biology, National Centre for Scientific Research “Demokritos”, Athens, Greece; 3 Inscent, Inc., Irvine, California, United States of America; 4 Department of Agricultural Biotechnology, Agricultural University of Athens, Athens, Greece; 5 Institute of Biology, Faculty of Science, University of Neuchâtel, Neuchâtel, Switzerland; 6 Joint Science Department of the Claremont Colleges, Claremont McKenna College, Pitzer College, and Scripps College, Claremont, California, United States of America; Pennsylvania State University, United States of America

## Abstract

Haematophagous insects are frequently carriers of parasitic diseases, including malaria. The mosquito *Anopheles gambiae* is the major vector of malaria in sub-Saharan Africa and is thus responsible for thousands of deaths daily. Although the role of olfaction in *A. gambiae* host detection has been demonstrated, little is known about the combinations of ligands and odorant binding proteins (OBPs) that can produce specific odor-related responses *in vivo*. We identified a ligand, indole, for an *A. gambiae* odorant binding protein, AgamOBP1, modeled the interaction *in silico* and confirmed the interaction using biochemical assays. RNAi-mediated gene silencing coupled with electrophysiological analyses confirmed that AgamOBP1 binds indole in *A. gambiae* and that the antennal receptor cells do not respond to indole in the absence of AgamOBP1. This case represents the first documented instance of a specific *A. gambiae* OBP–ligand pairing combination, demonstrates the significance of OBPs in odor recognition, and can be expanded to the identification of other ligands for OBPs of *Anopheles* and other medically important insects.

## Introduction

The mosquito *Anopheles gambiae* is the major sub-Saharan vector for the malaria parasite, *Plasmodium falciparum*. Female *A. gambiae* rely on their sense of smell for sugar feeding and oviposition [1], [2]as well as responding to human odors to find a blood meal[3]–[6]. Since olfaction is linked to crucial behaviors, understanding olfactory processes in more detail can lead to improved insect control strategies [7].

Volatile odorants are detected and discriminated by olfactory receptor neurons (ORNs) housed in sensory hairs, sensilla, which are located on the mosquito antennae as well as the maxillary palps. According to the current model of olfaction, odorants enter the sensillar lymph from the air through cuticular pores and are captured by odorant binding proteins (OBPs) that transport them through the sensillar lymph to odorant receptors (ORs) localized on the dendritic membranes of olfactory neurons. After stimulation by cognate ligands, ORs transduce the signals to downstream effector molecules [8].

OBPs and the structurally-related pheromone binding proteins (PBPs) are the first proteins to interact with the odor and, by an inherent binding preference determined by their ligand pocket that is formed by six α-helices [9], may help determine odor responses. Moreover, odor recognition is likely a coordinated process requiring the combined specificities contributed by OBPs and ORs and thus, optimal tuning and sensitivity of an olfactory sensillum would result when there is expression in the same sensillum of an OBP and an OR binding the same class of odor molecules [10]–[14]. Thus, OBPs are potentially key components of receptor cell specificity as defined by levels of sensitivity to specific odorants.

The A. gambiae genome contains about 60 putative OBP-encoding genes [15]–[17].Among them, AgamOBP1 has significantly elevated mRNA concentrations in female vs. male heads, and is down-regulated after a blood meal. Because of these characteristics, the suggestion was previously made that AgamOBP1 could be involved in host seeking behavior [18]. AgamOBP1 was also crystallized and its structure determined at a resolution of 1.5 Å [19], however a native ligand was not identified.

Here, we describe the identification of a ligand, indole, for AgamOBP1. RNAi-mediated gene silencing was used to attenuate the expression of AgamOBP1 *in vivo* and electrophysiological tests confirmed the ligand-OBP relationship by demonstrating that antennae from uninjected and AgamOBP7-dsRNA injected female mosquitoes respond to indole while antennae from females injected with AgamOBP1-dsRNA no longer do so. In previous studies, *A. gambiae* showed electroantennogram (EAG) responses to indole originating from human sweat [20] and to indole and 3-methyl indole as constituents of water in breeding sites in Tanzania [21]. Our data comprise the first instance of an *A. gambiae* OBP-ligand pairing and the first time that electrophysiology and dsRNA-mediated inhibition of gene expression are combined to confirm a ligand recognition pathway mediated by a specific OBP in mosquitoes. These results demonstrate the importance of OBPs in the control of odor responses and delineate a general approach for analyzing olfactory-mediated behavior in medically important insects.

## Results

### Screening for Binding of Natural Ligands to AgamOBP1

**Figure 1:**
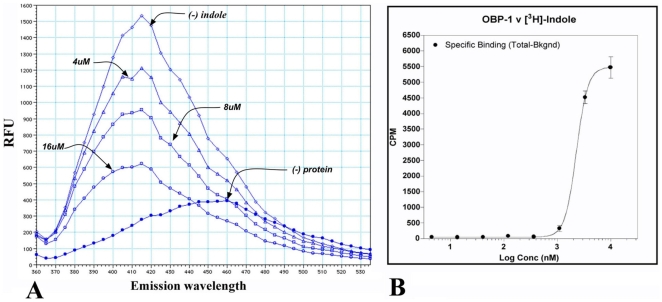
Fluorescence screening system for the detection of native ligands for OBPs. Three concentrations of each ligand (16 µM, 8 µM and 4 µM) were tested using 4 µM of r-OBP1 (left panel). Indole was found to bind r-OBP1 as increasing concentrations of indole displaced more dye from the AgamOBP1 binding pocket. (right panel) Binding curve of ^3^H indole to r-OBP1 in Ni-NTA FlashPlates. Specific binding was determined after background subtraction from uncoated wells. Curve fitting was performed with GraphPad Prism and the calculated K_D_ is 2.3 µM±0.3 (95 % CI).

Three recombinant *A. gambiae* OBPs (r-OBPs), r-OBP1, r-OBP20 and r-OBP48 were examined for their binding capacities of 22 putative ligands known to elicit EAG responses in *A. gambiae* females (Table S1). The screening assays, an example of which is shown in Fig. 1, have revealed that of these putative ligands, only indole bound to the recombinant OBP1 (r-OBP1). Furthermore, the indole derivative, 3-methyl indole (skatole), a well-characterized oviposition stimulant for *A. gambiae* [21] and *Culex spp* mosquitoes [22]–[27], also bound to r-OBP1 but with an apparently lower affinity. The two other recombinant *A. gambiae* OBPs (r-OBP20 and r-OBP48) failed to show significant binding to any of the tested compounds. Indole binding to r-OBP1 was confirmed *in vitro* using radiolabeled ligands in a scintillation proximity assay (SPA; Fig. 1). We observed a very steep response over a small concentration range of indole, which is indicative of biological significance for odor binding to an OBP. The calculated Kd of 2.3 µM is within the range of binding of pheromones (0.1–7.1 µM) and other ligands (0.14–6.2 µM) to insect PBPs and OBPs [9].

### Modeling of AgamOBP1 with Indole as Ligand

**Figure 2:**
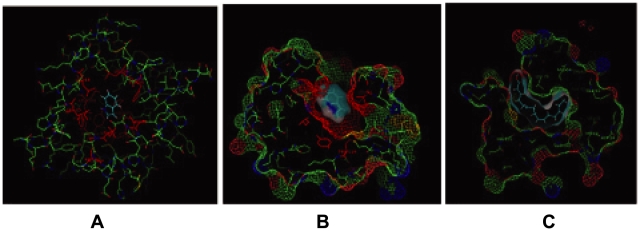
*In silico* ligand binding studies on *Anopheles gambiae* OBP1. (A) AgamOBP1 with indole fitted in the binding cavity. Cyan is the indole ring. Residues in red line the binding site. (B) Surface representation of the AgamOBP1 binding cavity (wired mesh) and indole (semi transparent continuous surface) showing the calculated binding position of indole fitted in the upper part of the binding site. (C) Predicted oleic acid (cyan) binding position indicating the L shaped binding cavity. Diagrams created with PYMOL (DeLano Scientific LLC).

Ligand binding to AgamOBP1 was modeled in accordance with the protein structure previously described [19]. The binding pocket (Fig. 2) has an elongated cylindrical shape lined mainly with hydrophobic residues (Leu15, Leu19, Leu58, Phe59, Leu76, Leu80, Met84, Leu124) and other residues possessing polar properties (His111, Trp114, Tyr122; numbering of PDB ID: 2ERB). The binding cavity is L-shaped with similarities to honeybee PBP [28] the wider part being towards the rim of the entrance. The pocket is wide enough to accommodate flat double ring structures such as indole (Fig. 2A and B). The binding cavity can also accommodate elongated, mainly hydrophobic molecules without long side chains, such as oleic acid (Fig. 2C). The fact that the crystal structure of an AgamOBP1 dimer has been determined in the presence of the very long PEG molecule occupying both binding sites of the monomers through a polar dimer interface may indicate the ability of the ligand binding pocket to accommodate various sizes and types of ligands. Apart from imposed shape constraints on the pocket from the mainly hydrophobic lining, side chains and the presence of very few polar residues, no apparent ligand discrimination through side chain interaction is evident.

### Specific Reduction of OBP Gene Expression in Antennae by RNAi

We used RNAi to attenuate expression of specific *A. gambiae* antennal OBPs and applied this approach to investigate whether OBPs mediate odor perception *in vivo*. dsRNA is stable for several days after injection into adult mosquitoes and can provide a long-lasting inhibition of endogenous gene expression [29]–[31].

**Figure 3:**
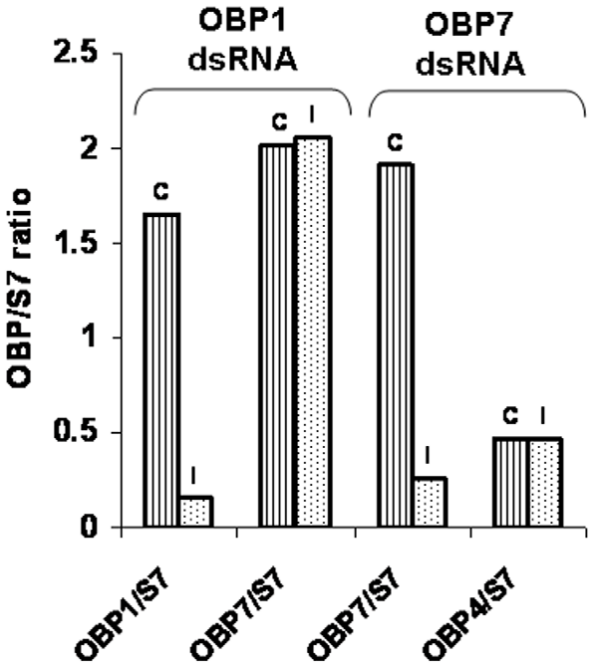
qRT-PCR determination of mRNA levels in mosquitoes. Pools of 5 female mosquitoes 4 days after injection with AgamOBP1-dsRNA or AgamOBP7-dsRNA were analysed. Values were normalized to RpS7(S7). While OBP mRNA levels of a control OBP (AgamOBP7-left, AgamOBP4-right) remained unchanged, mRNA levels of the corresponding injected dsRNA (AgamOBP1-left, AgamOBP7-right) were reduced about 10-fold; C control, I injected.

The sequences of *A. gambiae* OBPs differ sufficiently so that no cross-interference was expected. Indeed, injection of AgamOBP1-dsRNA reduced significantly the concentration of AgamOBP1 mRNA levels but did not alter AgamOBP7 or AgamOBP48 mRNA levels (Fig. 3 and Table S2). Because of possible variations between injected individuals, RNA was isolated from pools of five AgamOBP1-dsRNA-injected mosquitoes, converted to cDNA and analyzed by qRT-PCR. Variable but significant reduction of the AgamOBP1 mRNA was detected in ds-RNA injected mosquitoes (Table S2). Ten-fold reductions were observed routinely. Likewise, injection of AgamOBP7-dsRNA reduced AgamOBP7 mRNA levels ∼10 fold but did not alter AgamOBP1, AgamOBP4 and AgamOBP48 mRNA levels (Fig. 3 and Table S3). These results establish the feasibility of using RNAi for inhibition of OBP gene expression in the antennae of *A. gambiae* to validate OBP target specificity.

**Figure 4:**
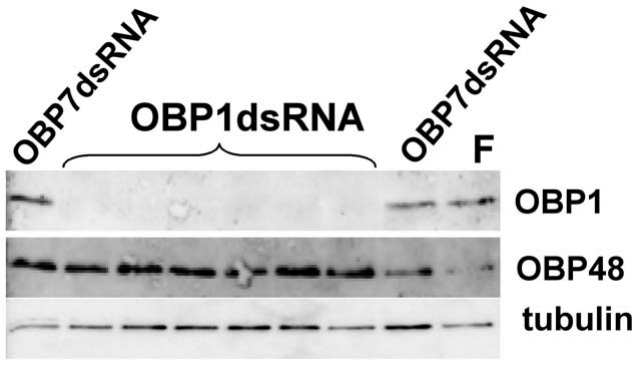
Western blot analyses for the detection of OBP1. Individual head extracts of AgamOBP1-dsRNA-injected female mosquitoes (OBP1dsRNA), as well as AgamOBP7-dsRNA-injected (OBP7dsRNA) or uninjected females (F) (top panel) were subjected to SDS PAGE and Western blot. The membranes were subsequently incubated, without stripping, with an anti-AgamOBP48 antibody (middle panel) and, finally, after stripping, with an anti-tubulin antibody (lower panel).

To investigate whether AgamOBP1 protein levels are also reduced in response to AgamOBP1-dsRNA injections, we extracted proteins from individual heads of females that had been injected 4 days earlier with AgamOBP1 or AgamOBP7-ds RNA and from uninjected females serving as controls, and examined them for the presence of AgamOBP1 and AgamOBP48 in western blot assays. While AgamOBP1 was consistently and easily detectable in the heads of uninjected females and females that had been injected with AgamOBP7-ds RNA (Fig. 4), it could not be detected in any of the heads of females that had been injected with AgamOBP1-ds RNA. It is also worth noting that, in general, no differences could be observed in the levels of OBP48 accumulation between the heads of the control females and those that had been injected with either AgamOBP1- or AgamOBP7-ds RNA. These findings establish both the effectiveness of down regulation of *obp* gene expression of choice in the antennae of *A. gambiae* and the specificity of the silencing process. Moreover, these results demonstrate that the turnover of AgamOBP1 mRNA is sufficiently fast that, at 4 days after dsRNA injection, protein levels are significantly lowered, consistent with the reduced mRNA levels.

### Electrophysiological Recordings from dsRNA-Injected Females

**Figure 5:**
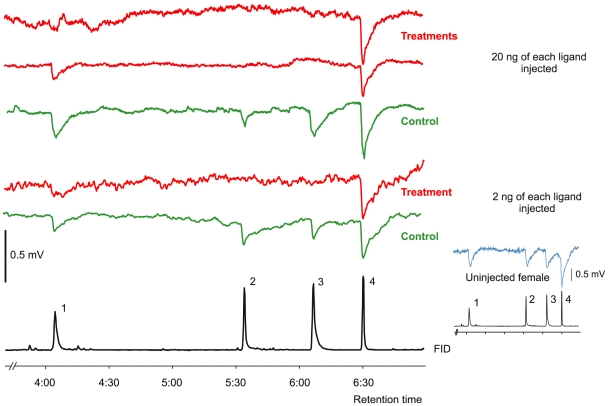
Electroantennogram responses of *Anopheles gambiae* females. Electroantennogram responses of *Anopheles gambiae* females to p-cresol (1), indole (2), 3-methyl indole (3) and geranylacetone (4) eluting from an apolar gas chromatographic column. For the top 3 traces, 20 ng quantities of p-cresol, indole, 3-methyl indole and geranylacetone were injected and for the next two traces down 2 ng of these ligands were injected. The column effluent was split (50:50) between the flame ionization detector (FID, bottom trace) of the chromatograph and the antennal preparations (control and treatments above). In recordings from the antennae of three females injected with AgamOBP1-dsRNA (treatments) the response to indole and 3-methyl indole was silenced whereas in two recordings from the antennae of a females injected with AgamOBP7-dsRNA (controls) the responses to indole and 3-methyl indole were no different to that of an uninjected female (small insert on right); mV scale common to treatment and control recordings.

To establish that AgamOBP1 knockdown by RNAi causes a significant reduction in *A. gambiae* electrophysiological responses to indole, EAG responses to geranylacetone, p-cresol, indole and 3-methyl-indole were recorded from control and dsRNA-treated *A. gambiae* females (Fig. 5). Results obtained with indole and the structurally related 3-methyl indole were compared to the responses with geranylacetone, to which the *A. gambiae* antennae are sensitive at a level below 20 ng when delivered by gas chromatography. The EAG response to geranylacetone was always the strongest in all mosquitoes with the absolute response to this product varying up to 2-fold or higher between individuals. Despite this variation, a near complete knockdown in the relative response to indole was recorded in the AgamOBP1-dsRNA-treated mosquitoes. The EAG responses of uninjected mosquitoes were 41% for indole, 14% for 3-methyl-indole and 57% for p-cresol relative to geranylacetone (Table 1) whereas the EAG responses from females injected with 500–800 ng of AgamOBP1-dsRNA showed a significant reduction in responses to indole and 3-methyl indole (Table 2). Compared to EAG recordings from uninjected mosquitoes there was complete knockdown of the response to indole in 7 of 9 female antennae, and in 6 of these individuals no response to 3-methyl indole could be recorded either. The relative EAG responses to p-cresol were also decreased in mosquitoes treated with AgamOBP1-dsRNA but the median EAG level was not different from control mosquitoes. Mosquitoes injected with 600 ng of AgamOBP7-dsRNA were also analyzed, but no change was recorded in their EAG responses to any of the four ligands tested.

**Table 1:** EAG responses of *Anopheles gambiae* females to the olfactory stimulants indole, 3-methylindole and *p*-cresol (normalized with respect to the responses to geranylacetone).

Female *A. gambiae* uninjected (control group)			
No	indole	3-methyl indole	p-cresol
	rel response	rel response	rel response
1	0.41	0.14	nt
2	0.18	0.08	nt
3	0.35	0.26	nt
4	0.39	0.00	nt
5	0.56	0.11	0.67
6	0.57	0.30	0.40
7	0.57	0.37	0.57
8	0.33	0.08	0.42
9	0.20	0.00	0.29
10	0.62	0.62	1.08
11	0.41	0.41	1.06
**median**	**0.41**	**0.14**	**0.57**

**nt** not tested.

**Table 2:** EAG responses of ds RNA injected *Anopheles gambiae* females to the olfactory stimulants indole, 3-methylindole and *p*-cresol (normalized with respect to the responses to geranylacetone).

Female *A. gambiae*, 500 to 800 ng AgamOBP1-dsRNA injected			
No	indole	3-methyl indole	p-cresol
	rel response	rel response	rel response
1	0	0	0.40
2	0	0	na
3	0	0	0.40
4	0.5	0	0
5	0	0	0.47
6	0	na	na
7	0.23	0.38	0.54
8	0.58	0.42	0.92
9	0.00	0.00	0.71
**median**	0.00 **	0.00 **	**0.47 ns**

Responses of mosquitoes injected with AgamOBP1-dsRNA five days prior to recordings. For both indole and 3-methylindole median responses of AgamOBP1-injected mosquitoes were significantly lower than the control group (Table 1) (** P≤0.01) but not for *p*-cresol (**ns** not significant) using the Mann - Whitney U-Test; **na** not analysed, **nt** not tested.

## Discussion

As was long suspected and recently demonstrated by a number of relevant studies, OBPs are responsible for the first level of control of the olfactory responses [32], [33]. Indeed, a recent study documented that odor preferences and oviposition behavior are greatly influenced by specific OBPs in *Drosophila* [34]. Moreover, the observation that OBPs can shift the specificity of *Bombyx mori* pheromone receptor responses expressed in a human cell line [35] or in the *Drosophila* “empty neuron” system [36] provided further experimental evidence for the necessity of proper pairing between ORs and OBPs. More compelling evidence for the cooperation between OBPs and ORs comes from recent experiments in *Drosophila* in which perception of the volatile pheromone 11-cis vaccenyl acetate (cVA) has been studied. cVA stimulates a receptor cell in T1 sensilla on the male antennae. These sensilla co-express the odorant receptor Or67d and the OBP76a encoded by the gene *lush*. Loss of OBP76a in *lush* mutants results in insensitivity to cVA, even though they properly express all ORs [37]. Mutants that lack T1 sensilla also lack Or67d and do not respond to cVA, but the cVA response can be restored by ectopically expressing Or67d in other sensilla if these sensilla also co-express OBP76a. Conformational changes of OBP proteins upon ligand binding appear to alter their structure in such a way that the receptor can distinguish between the “loaded” and “empty” form of the OBP. In fact, mutations in OBP76a that mimic the conformational shift caused by ligand binding can activate the olfactory response in the absence of cVA [38].

In a given sensillum, OBPs may contribute to the specificity of odor reception by exhibiting a binding preference for certain odor molecules thus selecting which odors to transport. As is the case with the *in vitro* binding of indole to AgamOBP1 (Kd of 2.3 µM; Fig. 1), ligand-binding specificities for OBPs with dissociation constants in the µM range have been previously established [9]. While PBPs often show a high degree of specificity and can discriminate between closely related compounds, OBPs appear to recognize a broader spectrum of odorants, suggesting that odor molecules may be bound with high affinity by one class of OBPs and with a lower affinity by another class [9]. Despite these differences, the binding pockets of PBPs and OBPs are structurally similar and formed by six α-helices, stabilized by disulfide bridges between six cysteines [39]–[42].

We used the published AgamOBP1 structure [19] to model the binding of indole into its binding pocket. Molecular mechanics calculations show possible orientations of indole in the binding pocket (Fig. 2A) as well as the feasibility of 3-methyl-indole binding. Further *in silico* studies of AgamOBP1 binding to a variety of candidate ligands showed a preference for elongated cylindrical molecules with no side chains, but small flat ring structures can be accommodated in the binding site as well. Additionally, polar groups may also be accommodated in some regions of the binding cavity. Modeling of the AgamOBP1 dimers suggests that binding of two ligand molecules may occur readily. The steep response curves we observed in the FlashPlate assays are indicative of a cooperative ligand binding and therefore may represent the binding of indole to AgamOBP1 dimers and/or trimers [43].

The essential role of AgamOBP1 in the perception of indole was ultimately demonstrated in this study by the EAG responses of *A. gambiae* females subjected to AgamOBP1-dsRNA injections, which caused a drastic reduction in AgamOBP1 accumulation. Most of these mosquitoes showed complete loss of the EAG response to indole. As predicted from the ligand-binding and modeling studies, the EAG responses to 3-methyl indole were also affected in the same mosquitoes. The specificity of gene expression inhibition induced by the injection of females with AgamOBP1-dsRNA was demonstrated by the unaltered EAG responses of the mosquitoes to the terpene, geranylacetone. No loss of the EAG responses to indole and 3-methyl indole was recorded in control females injected with AgamOBP7-dsRNA. These females were shown to contain levels of AgamOBP1 mRNA and protein comparable to those of controls. These results constitute the first record of blocking olfactory perception of critical ligands in a mosquito and support the claim that OBPs are valuable targets for interference with the olfactory response in mosquitoes.

The molecular mechanism of olfaction in insects is complex, comprising numerous classes of proteins and effectors that interact in order to translate an external stimulus in the environment to a behavioral response in the insect [8]. Since olfactory stimuli or odorants drive specific insect behaviors such as mating, oviposition and feeding, isolating the particular components of the insect system responsible for odorant recognition and odorant transport to neuronal cell surfaces in order to initiate downstream signaling will allow a rational design to be adopted in the development of novel insect control products. Olfactory pathway components responsible for key behaviors are suitable control product targets [8]. The work discussed herein represents an instance of an odorant molecule being paired with a specific component of the *A. gambiae* olfactory system, AgamOBP1. It was also shown that the ligand-OBP pair elicits a specific electrophysiological response in *A. gambiae* antennae, indicating that indole is detectable by these mosquitoes, and that this detection is dependent on the presence of AgamOBP1. Correlating these findings with the behavioral effects of indole observed on *C. quinquefasciatus* [22] raises intriguing directions for future research: first, to identify particular odorants or chemical stimuli that utilize specific components of the chemosensory pathway including olfactory receptors that are differentially regulated in male and female mosquito antennae [18], [44], and thus build an odor recognition-to-behavior “map” for *A. gambiae*; second, to target those specific components of the odor recognition pathway(s) that control crucial behavior(s) in order to generate novel attractants, repellents, or other behavior alteration products that will make possible the interruption of either the mosquito's life cycle or the cycle of malaria transmission from mosquito to human. The molecular and electrophysiological techniques described here in combination with behavioral assays will facilitate identification of key stimuli and those protein or effector components of the *Anopheles* olfactory system responsible for their recognition.

## Materials and Methods

### Expression of Recombinant OBPs, *in Vitro* Binding Assays and Antibodies Production

PCR-amplified AgamOBP1 (AF437884), and AgamOBP48 (AF533512) cDNAs (17, 45) were cloned in pRSET and recombinant protein produced in *E. coli* BL21 Star (DE3)pLysS cells. AgamOBP20 (AY146727) was expressed in BTI-TN-5B1-4 lepidopteran cells (HighFive™ Invitrogen) as previously described[43]. Twenty putative ligands that evoke an electrophysiological antennal response [5], [6], [8], [46], [47], were used in ligand binding tests using an established fluorescence-quenching assay [9] adapted by Inscent Inc. (Irvine, CA) for high throughput. The assay uses a fluorescent dye that modifies its emission spectrum upon binding the insect OBP, generally yielding a notable increase in intensity and a shift in peak emission wavelength, from 460 nm to 416 nm. Subsequently, a ligand capable of binding the OBP via the protein's binding pocket will displace the dye and in doing so quenching fluorescence; these changes are observed with a spectrophotometer. The dye used was 8 µM N-Phenyl-1-Naphthylamine (1-NPN; CAS 90-30-2) and the fluorescence screening system utilized three concentrations of each ligand (16 µM, 8 µM and 4 µM) and 4 µM of r-OBP1. Indole was found to bind r-OBP1 as increasing concentrations of indole displaced more 1-NPN dye from the AgamOBP1 binding pocket. Fluorescence was detected using a Molecular Devices Gemini XPS spectrofluorometer (Sunnyvale, CA).

Indole binding to AgamOBP1 was validated using radiolabeled indole in FlashPlate competitive assays (Perkin Elmer) based on the principle of scintillation proximity (SPA). Briefly, purified 6xHis tagged r-OBP1 (100 µl of 25 µg/ml) was bound in the wells of the Nickel chelate FlashPlate. ^3^H indole [specific activity, 5.1 Ci/mmol; concentration, 330 µM, custom-synthesized by ViTrax (Placentia, CA) and HPLC purified to >99% radiochemical purity] was added to the wells starting at 10 µM with eight sequential 1∶3 dilution steps and incubated for 10 min. Determinations were done in triplicate and paralleled with non-r-OBP1-coated wells for background controls.

AgamOBPs (r-OBP1 and r-OBP48) were purified from the soluble fraction of lysate using the SwellGel Cobalt Chelated Disc system (Pierce Chemical) and used to raise antibodies in guinea pigs (Pocono Rabbit Farm and Laboratory, Inc., Canadensis, PA). Anti-OBP1 and anti-OBP48 immune sera and the corresponding preimmune sera from single guinea pigs were tested by immunoblotting.

### AgamOBP1 Modeling with Indole as Ligand

Three-dimensional (3D) modeling and *in silico* binding studies on OBP were based on the crystal structure of AgamOBP1 (PDB ID: 2ERB) [19] with ligand structures from the Cambridge Structural Database [48]. Computational binding studies of ligands with OBP were performed by the QUANTA-CHARMM program using the CHARMM force field by simultaneously optimizing ligand conformation and rigid body position. Derived models were checked for folding and packing errors using QUANTA-CHARMM [49] to arrive at a protein-ligand complex with no bad atom contacts and optimal side-chain conformation. Ligands were ranked using an energy function dominated by van der Waals interactions and orientation-dependent hydrogen bonding potential.

### *In Vivo* RNA Interference with Gene Expression (RNAi)

Double-stranded RNA (dsRNA) was synthesized from full-length AgamOBP PCR products (400–500 bp) using the Ambion MEGAscript RNAi Kit. Forward (L) and reverse (R) OBP-specific primers were designed with T7 promoter sequence overhangs (Table S4) and used to amplify by PCR the target OBP cDNA. The template was a cDNA pool that was itself prepared by reverse transcription of RNA extracted from 50–60 mosquito heads. This technique eliminates the plasmid linearization step and loss of yield due to transcription of undigested, circular plasmids. T7-OBP cDNA was cleaned and concentrated using the Zymo Research DCC kit and between 1 and 2 ug of cDNA were used for the subsequent transcription reaction. For increased yield and quality, the incubation time of this step was extended to six hours. Post-transcription purification steps mirrored those given by the MEGAscript RNAi kit manual. Yield and quality were assessed using gel electrophoresis on a 2% agarose gel and quantification using an Implen NanoPhotometer.

Sixty five to 100 nl of the dsRNA solution (equivalent to 520–800 ng) were injected laterally into the thoraces of 1 to 3 day-old adult *A. gambiae* females and males (G3 strain) using a drawn out capillary (1 mm o.d.) with a 35–40 µm tip aperture connected via Teflon tubing to a 50 µl syringe (Hamilton, Bonaduz, CH) mounted to a syringe pump (CMA 400, DMA Microdialysis AB, Solna, SE). Three to five days after injection, total RNA was isolated from injected and control pools of 4–5 adults using 500 µl of TRIzol Reagent (Gibco BRL Life Technologies, Rockville, MD). The RNA was dissolved in 17 µl water and converted to cDNA using standard methods.

cDNA thus generated was used for quantitative RT-PCR (BioRad iCycler iQ™ Real-Time PCR cycler, BioRad, Hercules, CA). Reactions were performed according to the manufacturer's instructions (SYBR Green, Invitrogen, Carlsbad, CA; Hotmaster Taq polymerase, Eppendorf AG, Hamburg, DE) using OBP-specific primer pairs (Table S4). In every plate, a control curve was generated with each primer pair, and data for each sample point were acquired in three technical replicates. Determinations of mRNA abundance were undertaken in either two or three replicates. The obtained values were averaged and normalized for each preparation with the concentration of ribosomal protein S7 (RpS7) mRNA serving as control.

### Western Blot Assays for Detection of AgamOBP1 and AgamOBP48 in Head Extract Preparations

Individual heads dissected from single control or dsRNA-injected mosquitoes were homogenized in 30–40 µl of 1X SDS-sample buffer (62mM Tris-HCl pH 6.8, 2% SDS, 10% glycerol, 0.002% bromphenol blue) using a Kontes pellet pestle, followed by heating at 70°C for 10 min. After addition of 10% β-mercaptoethanol, proteins were separated on 15% SDS-PAGE gels and subsequently transferred to Hybond nitrocellulose membranes. Immunoblotting was initially performed with anti-OBP1 antibody at a 1∶800 dilution and a 1∶1,000 anti-guinea pig secondary antibody (Jackson ImmunoResearch Laboratories, Inc.), using the Amersham ECL Western Blotting detection reagents (GE Healthcare) or Pierce SuperSignal West Pico chemiluminescent substrate (ThermoScientific). Membranes were subsequently incubated, without stripping, with anti-OBP48 antibody at a 1∶1,000 dilution and a 1∶1,000 anti-guinea pig secondary antibody. Finally, after stripping, western blotting was performed with an anti-tubulin antibody (AbD Serotec) at a 1∶500 dilution, using an 1∶1,000 anti-rat secondary antibody (Chemicon, Millipore).

### Determination of Electrophysiological Responses to Natural Ligands

*Anopheles gambiae* (Giles) *ss* strain 16CSS were reared in a climate chamber at 80% RH and 28°C with 10∶10 h L/D photoperiod with 2 h light ramps at dawn and dusk with access *ad libitum* to 10% sucrose. One to 3 day-old female mosquitoes were injected with dsRNA as described above and subjected to electroantennogram (EAG) recordings 3–5 days later.

For EAG recordings, the head of each 4–8 day old control or dsRNA-injected female *A. gambiae* was excised at the occipital opening and placed on the reference glass electrode containing Hayes mosquito Ringer solution [51], mounted in a humidified air-stream (90–98% RH) and the antennae exposed to test ligands eluting from a gas chromatographic (GC) column [52]. The EAG response was recorded via a glass electrode filled with Kaissling sensillum lymph Ringer solution [53] brought into contact with the terminal antennal segment whose tip was cut off. Only antennae showing a response at least double the noise level to a puff of air over 1 µg geranylacetone in a 5 ml syringe were used for recording EAG responses to the ligand mixture. A mixture containing 100 to 500 ng geranylacetone, p-cresol, indole and 3 methyl indole (all from Fluka, Switzerland, >98% pure) was injected in splittless mode on-column in 1-3 µl methylene chloride onto a high-resolution capillary column with either a free-fatty-acid-phase (FFAP, modified polyethylene glycol phase esterified with terephthalic acid, 30 m long, 0.25 mm i.d., 0.25 µm film thickness for treatments with uninjected controls) or an apolar phase (95% dimethylpolysiloxan with 5% diphenylpolysiloxan, 15 m long, 0.25 mm i.d., 0.10 µm film thickness for treatments with AgamOBP7-dsRNA injected controls; both columns from BGB Analytik, Switzerland) installed in a 5300 Carlo Erba Instruments chromatograph. H2 was used as carrier gas and the oven was held at 40°C for 3 min then heated at 25°C/min to 230°C and held for 15 min. Relative responses to the four stimuli eluting from the GC column were normalized with respect to the strongest stimulus, geranylacetone. Differences of median EAG responses were analyzed with S-Plus (V6.2, build 6713, Insightful, Seattle, WA).

## Supporting Information

Table S1 List of compounds tested for OBP binding.
(0.02 MB DOC)

Table S2 Reduction of AgamOBP1 mRNA levels after injection of its corresponding dsRNA.
(0.03 MB DOC)

Table S3 Reduction of AgamOBP7 mRNA levels after injection of its corresponding dsRNA
(0.02 MB DOC)

Table S4 List of primers utilized for cDNA amplification, ds-RNA synthesis and qRT-PCR studies.
(0.02 MB DOC)

## References

[1] Takken W, Knols BG (1999). Odor-mediated behavior of Afrotropical malaria mosquitoes.. Annu Rev Entomol.

[2] Foster WA (1995). Mosquito sugar feeding and reproductive energetics.. Annu Rev Entomol.

[3] Dekker T, Takken W, Braks MA (2001). Innate preference for host-odor blends modulates degree of anthropophagy of *Anopheles gambiae* sensu lato (Diptera: Culicidae).. J Med Entomol.

[4] Dekker T, Steib B, Carde RT, Geier M (2002). L-lactic acid: a human-signifying host cue for the anthropophilic mosquito *Anopheles gambiae*.. Med Vet Entomol.

[5] Costantini C, Birkett MA, Gibson G, Ziesmann J, Sagnon NF (2001). Electroantennogram and behavioral responses of the malaria vector *Anopheles gambiae* to human-specific sweat components.. Med Vet Entomol.

[6] Meijerink J, Braks MA, Van Loon JJ (2001). Olfactory receptors on the antennae of the malaria mosquito *Anopheles gambiae* are sensitive to ammonia and other sweat-borne components.. J Insect Physiol.

[7] Justice RW, Biessmann H, Walter MF, Dimitratos SD, Woods DF (2003). Genomics spawns novel approaches to mosquito control.. Bioessays.

[8] Hallem EA, Dahanukar A, Carlson JR (2006). Insect odor and taste receptor Annu Rev Entomol.

[9] Pelosi P, Zhou JJ, Ban LP, Calvello M (2006). Soluble proteins in insect chemical communication.. Cell Mol Life Sci.

[10] Grosse-Wilde E, Svatos A, Krieger J (2006). A pheromone-binding protein mediates the bombykol-induced activation of a pheromone receptor *in vitro*.. Chem Senses.

[11] van der Goes van Naters W, Carlson JR (2007). Receptors and neurons for fly odors in *Drosophila*.. Curr Biol.

[12] Ha TS, Smith DP (2006). A pheromone receptor mediates 11-cis-vaccenyl acetate-induced responses in *Drosophila*.. J Neurosci.

[13] Vogt RG, Prestwich GD, Lerner MR (1991). Odorant-Binding-Protein subfamilies associate with distinct classes of olfactory receptor neurons in insects.. J Neurobiology.

[14] Vogt RG (2003). Biochemical diversity of odor detection: OBPs, ODEs and SNMPs in *Insect Pheromone Biochemistry and Molecular Biology*..

[15] Vogt RG (2002). Odorant binding protein homologues of the malaria mosquito *Anopheles gambiae*; possible orthologues of the OS-E and OS-F OBPs of *Drosophila melanogaster*.. J Chem Ecol.

[16] Xu PX, Zwiebel LJ, Smith DP (2003). Identification of a distinct family of genes encoding atypical odorant-binding proteins in the malaria vector mosquito, *Anopheles gambiae*.. Insect Mol Biol.

[17] Biessmann H, Nguyen QK, Le D, Walter MF (2005). Microarray-based survey of a subset of putative olfactory genes in the mosquito *Anopheles gambiae*.. Insect Mol Biol.

[18] Justice R W, Dimitratos S, Walter M F, Woods D F, Biessmann H (2003). Sexual dimorphic expression of putative antennal carrier protein genes in the malaria vector Anopheles gambiae.. Insect Mol Biol.

[19] Wogulis M, Morgan T, Ishida Y, Leal WS, Wilson DK (2006). The crystal structure of an odorant binding protein from *Anopheles gambiae*: evidence for a common ligand release mechanism.. Biochem Biophys Res Commun.

[20] Meijerink J, Braks MAH, Brack AA, Adam W, Dekker T (2000). Identification of olfactory stimulants for *Anopheles gambiae* from human sweat samples.. J Chem Ecol.

[21] Blackwell A, Johnson SN (2000). Electrophysiological investigation of larval water and potential oviposition chemo-attractants for *Anopheles gambiae* s.s.. Ann Trop Med Parasitol.

[22] Millar JG, Chaney JD, Mulla MS (1992). Identification of oviposition attractants for *Culex quinquefasciatus* from fermented Bermuda grass infusions.. J Am Mosq Control Assoc.

[23] Blackwell A, Mordue (Luntz) AJ, Hansson B, Wadhams LJ, Pickett JA (1993). A behavioral and electrophysiological study of oviposition cues for *Culex quinquefasciatus*.. Physiol Entomol.

[24] Mordue (Luntz) AJ, Blackwell A, Hansson B, Wadhams LJ, Pickett JA (1992). Behavioral and physiological evaluation of oviposition attractants from *Culex quinquefasciatus* say (Diptera: Culicidae).. Experientia.

[25] Olagbemiro TO, Birkett MA, Mordue (Luntz) AJ, Pickett JA (2004). Laboratory and field responses of the mosquito, *Culex quinquefasciatus*, to plant-derived Culex spp. oviposition pheromone and the oviposition cue skatole.. J Chem Ecol.

[26] Leal WS, Barbosa RM, Xu W, Ishida Y, Syed Z (2008). Reverse and conventional chemical ecology approaches for the development of oviposition attractants for Culex mosquitoes.. PLoS One.

[27] Ishida Y, Cornel AJ, Leal WS (2002). Identification and cloning of a female antenna-specific odorant-binding protein in the mosquito *Culex quinquefasciatus*.. J Chem Ecol.

[28] Lartigue A, Gruez A, Briand L, Blon F, Bézirard V (2004). Sulfur single-wavelength anomalous diffraction crystal structure of a pheromone-binding protein from the honeybee *Apis mellifera L*.. J Biol Chem.

[29] Blandin S, Moita LF, Köcher T, Wilm M, Kafatos FC (2002). Reverse genetics in the mosquito *Anopheles gambiae*: targeted disruption of the Defensin gene.. EMBO Rep.

[30] Abraham EG, Donnelly-Doman M, Fujioka H, Ghosh A, Moreira L (2005). Driving midgut-specific expression and secretion of a foreign protein in transgenic mosquitoes with AgAper1 regulatory elements.. Insect Mol Biol.

[31] Moita LF, Wang-Sattler R, Michel K, Zimmermann T, Blandin S (2005). In vivo identification of novel regulators and conserved pathways of phagocytosis in *A. gambiae*.. Immunity.

[32] Vogt RG, Riddiford LM (1981). Pheromone binding and inactivation by moth antennae.. Nature.

[33] Vogt RG, Riddiford LM Pretwich GD (1985). Kinetic poperties of a pheromone degrading enzyme: the sensillar esterase of *Antheraea polyphemmus*.. Proc Natl Acad Sci USA.

[34] Matsuo T, Sugaya S, Yasukawa J, Aigaki T, Fuyama Y (2007). Odorant-binding proteins OBP57d and OBP57e affect taste perception and host-plant preference in *Drosophila sechellia*.. PLoS Biol.

[35] Grosse-Wilde E, Svatos A, Krieger J (2006). A Pheromone-Binding Protein Mediates the Bombykol-Induced Activation of a Pheromone Receptor in vitro.. Chem Senses.

[36] Syed Z, Ishida Y, Taylor K, Kimbrell DA, Leal WS (2006). Pheromone reception in fruit flies expressing a moth's odorant receptor.. Proc Natl Acad Sci USA.

[37] Xu P, Atkinson R, Jones DN, Smith DP (2005). *Drosophila* OBP LUSH is required for activity of pheromone-sensitive neurons.. Neuron.

[38] Laughlin JD, Soo T H, Jones DNM, Smith DP (2008). Activation of pheromone-sensitive neurons is mediated by conformational activation of pheromone-binding protein.. Cell.

[39] Tegoni M, Campanacci V, Cambillau C (2004). Structural aspects of sexual attraction and chemical communication in insects.. Trends Biochem Sci.

[40] Sandler BH, Nikonova L, Leal WS, Clardy J (2000). Sexual attraction in the silkworm moth: structure of the pheromone-binding-protein-bombykol complex.. Chem Biol.

[41] Klusak V, Havlas Z, Rulisek L, Vondrasek J, Svatos A (2003). Sexual attraction in the silkworm moth: nature of binding of bombykol in pheromone binding protein-an ab initio study.. Chem Biol.

[42] Mohl C, Breer H, Krieger J (2002). Species-specific pheromonal compounds induce distinct conformational changes of pheromone binding protein subtypes from *Antheraea polyphemus*.. Invert Neurosci.

[43] Andronopoulou E, Labropoulou V, Douris V, Woods DF, Biessmann H (2006). Specific interactions among odorant-binding proteins of the African malaria vector *Anopheles gambiae*.. Insect Mol Biol.

[44] Iatrou K, Biessmann H (2008). Sex-biased expression of odorant receptors in antennae and palps of the African malaria vector *Anopheles gambiae*.. Insect Biochem Mol Biol.

[45] Biessmann H, Walter MF, Dimitratos S, Woods DF (2002). Isolation of cDNA clones encoding putative odorant binding proteins from the antennae of the malaria-transmitting mosquito, *Anopheles gambiae*.. Insect Mol Biol.

[46] van den Broek, den Otter CJ (1999). Olfactory sensitivities of mosquitoes with different host preferences (*Anopheles gambiae* s.s., *An. arabiensis*, *An. quadriannulatus*, *An. m. atroparvus*) to synthetic host odours.. J Insect Physiol.

[47] Meijerink J, van Loon JJ (1999). Sensitivities of antennal olfactory neurons of the malaria mosquito, *Anopheles gambiae*, to carboxylic acids.. J Insect Physiol.

[48] Allen F (2002). The Cambridge Structural Database a quarter of a million crystal structures and rising.. Acta Crystallographica.

[49] Brooks BR, Bruccoleri RE, Olafson BD, States DJ, Swaminathan S (1983). CHARMM a program for macromolecular energy minimization and dynamics calculations.. J Comp Chem.

[50] Fujita Y, Kasuya A, Matsushita Y, Suga M, Kizuka M (2005). Structural elucidation of A-74528, an inhibitor for 2′,5′-phosphodiesterase isolated from *Streptomyces sp*.. Bioorg Med Chem Lett.

[51] Hayes RO (1953). Determination of a physiological saline for *Aedes aegypti* (L.).. J Econ Entomol.

[52] Arn H, Stadler E, Rauscher S (1975). The electroantennographic detector - a selective and sensitive tool in gas chromatographic analysis of insect pheromones.. Z Naturforsch.

[53] Kaissling K-E (1995). Single unit and electroantennogram recordings in insect olfactory organs..

