# Supplementary material for: The Anopheles gambiae Odorant Binding Protein 1 (AgamOBP1) Mediates Indole Recognition in the Antennae of Female Mosquitoes
Source: PLoS One. 2010 Mar 1;5(3):e9471. doi: 10.1371/journal.pone.0009471 (PMC2830424; doi:10.1371/journal.pone.0009471)
Supplement: Table S1 — List of compounds tested for OBP binding. (0.02 MB DOC) [file pone.0009471.s001.doc]

| L-lactic acid | formic acid | 6-methyl-5-hepten-2-one |
| --- | --- | --- |
| propionic acid | n-butyric acid | indole |
| heptanoic acid | n-valeric acid | pentylamine |
| 1-octen-3-ol | caproic acid | oleic acid |
| geranylacetone | octanoic acid | stearic acid |
| 3-methyl-1-butanol | p-cresol | indole-2-carboxylic acid |
| butylamine | m-cresol | 1-methyl indole |
|  |  | 5-methyl indole |

**Table S1.** List of compounds tested for OBP binding.
