# Supplementary material for: The Anopheles gambiae Odorant Binding Protein 1 (AgamOBP1) Mediates Indole Recognition in the Antennae of Female Mosquitoes
Source: PLoS One. 2010 Mar 1;5(3):e9471. doi: 10.1371/journal.pone.0009471 (PMC2830424; doi:10.1371/journal.pone.0009471)
Supplement: Table S2 — Reduction of AgamOBP1 mRNA levels after injection of its corresponding dsRNA. (0.03 MB DOC) [file pone.0009471.s002.doc]

| **Experiment 1** | | | | **Experiment 2** | | | |
| --- | --- | --- | --- | --- | --- | --- | --- |
|
|  | **OBP1** | **OBP7** | **OBP48** |  | **OBP1** | **OBP7** | **OBP48** |
| Female pool A | **10 ± 6**  (8.5; 17.1; 5.4 ) | **0.7** | **1.5** | Female pool A | **6** (6.0; 6.7 ) | **1.3** | **nd** |
| Female pool B | **15**  (12.4; 17.1) | **1.0** | **0.6** | Female pool B | **28**  (24.9; 30.6) | **1.1** | **nd** |

**Table S2:** Reduction of AgamOBP1 mRNA levels after injection of its corresponding dsRNA in the thoraces of mosquitoes. Numbers indicate the fold-reduction of mRNA levels of the injected versus control mosquitoes (injected with water) for several different OBPs, relative to the unchanged ribosomal protein S7 (RpS7) control mRNA. Two identical experiments were performed and the results shown separately to illustrate the variability in the levels of gene expression knockdown. From each experiment, injected mosquitoes were pooled in groups of 5 and RNA was extracted from each pool at 4 days after dsRNA injection. qRT-PCR determination of AgamOBP1, AgamOBP7 and AgamOBP48 specific mRNA levels was performed for 2 female pools in each experiment. Numbers in bold are the averages of multiple determinations of gene knockdown values (fold reduction) for each mosquito pool, and standard deviations are presented when appropriate. Multiple numbers in parenthesis represent data from independent qRT-PCRs performed with RNA extracted from the same dsRNA-injected pool of mosquitoes. Nd=not determined.
