# Supplementary material for: The Anopheles gambiae Odorant Binding Protein 1 (AgamOBP1) Mediates Indole Recognition in the Antennae of Female Mosquitoes
Source: PLoS One. 2010 Mar 1;5(3):e9471. doi: 10.1371/journal.pone.0009471 (PMC2830424; doi:10.1371/journal.pone.0009471)
Supplement: Table S3 — Reduction of AgamOBP7 mRNA levels after injection of its corresponding dsRNA (0.02 MB DOC) [file pone.0009471.s003.doc]

|  | **OBP7** | **OBP1** | **OBP4** | **OBP48** |
| --- | --- | --- | --- | --- |
| Female pool 1 | **8.6**  (11.2; 6.0) | **1** | **1** | **0.94** |
| Female pool 2 | **5.1** (5.6; 4.5) | **0.6** | **0.7**  (0.6; 0.8) | **nd** |

**Table S3**. Reduction of AgamOBP7 mRNA levels after injection of its corresponding dsRNA in the thoraces of mosquitoes. Numbers indicate the gene expression knockdown effects (fold-reduction of mRNA levels) of the injected versus control mosquitoes (injected with water) for several different OBPs, relative to the unchanged ribosomal protein S7 (RpS7) control mRNA. Details of the experiments and presentation of data are as described in Table S2.
