# Supplementary material for: The Anopheles gambiae Odorant Binding Protein 1 (AgamOBP1) Mediates Indole Recognition in the Antennae of Female Mosquitoes
Source: PLoS One. 2010 Mar 1;5(3):e9471. doi: 10.1371/journal.pone.0009471 (PMC2830424; doi:10.1371/journal.pone.0009471)
Supplement: Table S4 — List of primers utilized for cDNA amplification, ds-RNA synthesis and qRT-PCR studies. (0.02 MB DOC) [file pone.0009471.s004.doc]

| **Primer ID** | **Sequence 5'-3'** |
| --- | --- |
| RibS7-L | GCAGACCACCATCGAACAC |
| RibS7-R | GCTGCAAACTTCGGCTATTC |
| OBP1-L | TGTTACTGAGGAAGCGATCAAGAAG |
| OBP1-R | TGCAGCTTCTCCAGATGCAC |
| OBP4-L | TGGCAGGAACTATGACGAAGAAG |
| OBP4-R | AAAATCGGCGGCACATTTAG |
| OBP7-L | GCGAATGTAGCCACATCGTAAC |
| OBP7-R | GGTAAAACAAACACATTGGCAAC |
| T7OBP7-L | TAATACGACTCACTATAGGGTACATGTGTGAATATTCGAATACG |
| T7OBP7-R | TAATACGACTCACTATAGGGTACTCTCCAGCACTAGTAGGTGG |
| T7OBP4-L | TAATACGACTCACTATAGGGTACTATGAGCGTATCGGTGCTG |
| T7OBP4-R | TAATACGACTCACTATAGGGTACTTGGGAACATGAAGGTGTCG |
| T7OBP1-L | TAATACGACTCACTATAGGGTACTATGAAGCTAGTGACCTTCG |
| T7OBP1-R | TAATACGACTCACTATAGGGTACTCACTAAGAAATAGTGCTTCGG |

**Table S4**. List of primers utilized for cDNA amplification, ds-RNA synthesis and qRT-PCR studies.
